# Reasonable adjustments for autistic adults

**DOI:** 10.1192/bjo.2021.253

**Published:** 2021-06-18

**Authors:** Nazish Hashmi, Conor Davidson

**Affiliations:** Leeds and York NHS foundation trust

## Abstract

**Aims:**

To embed the use of reasonable adjustments for adults with autism within in mental health services.

**Objectives:**

The objectives of the project are as follows:

- To identify how many service users with a diagnosis of autism are under care of local mental health services
- Is there evidence that reasonable adjustments were considered for these service users
- If identified as needing reasonable adjusments is there evidence of such adjustments being made

**Method:**

We looked at service users with an established diagnosis of autism under care of Leeds and York NHS foundation trust to ascertain if reasonable adjustments have been considered. The audit is based on guidelines provided by Think Autism-department of health statutory guidance 2014. This is based on autism act 2009.

Data were collected for 30 cases in mainstream mental health services undr care of various teams including inpatient and community.

**Result:**

It was identified that in only 2/30 cases reasonable adjustments were considered and agreed upon. Only 1/30 service users had a disability status updated on electronic patient records. None of the service users had a hospital passport or reasonable adjustment care plan completed.None of the records had “good evidence” of reasonable adjustments.

These findings point to a wider issue for the trust as well as natioanlly as it indicates that autism is not being adequately taken into account for patients accessing our services. Due to the lack of reasonable adjustments adults with autism are potentially at increased risk to disengage leading to deterioration in their mental state and increase in risks.

**Conclusion:**

These findings point to a wider issue for the trust as it indicates that autism is not being adequately taken into account for patients accessing our services. Due to the lack of reasonable adjustments adults with autism are potentially at increased risk to disengage leading to deterioration in their mental state and increase in risks.

We recommend training in autism for all healthcare professionals in the trust to improve their understanding of autism, including making reasonable adjustments.

We also recommend review trust procedure about recording diagnoses and disability status on electronic patient records. We recommend that the reasonable adjustments section on care director is more prominent and easily accessible.

We recommend that an ‘autism flag’ is prominent on patient records to alert staff to the presence of autism

